# Reply: molecular determinants of prognosis in oesophageal cancer patients treated with chemoradiation

**DOI:** 10.1038/sj.bjc.6604647

**Published:** 2008-09-02

**Authors:** R Yoshikawa, M Sasako, Y Fujiwara

**Affiliations:** 1 Department of Genetics, Hyogo College of Medicine, 1-1, Mukogawa-cho, Nishinomiya, Hyogo 663-8501, Japan; 2 Department of Surgery, Hyogo College of Medicine, 1-1, Mukogawa-cho, Nishinomiya, Hyogo 663-8501, Japan

**Sir,**

We thank Dr Ajani and colleagues for their interest in our study and apologize for not citing their earlier study (Sims-Mourtada *et al*, 2006) in our original paper. From the aspect of management for metastasis in treatment-failure patients, we designed our study to evaluate the clinical implications of Hedgehog (Hh) signal activation for oesophageal squamous cell carcinoma (ESCC) patients who underwent pre-operative chemoradiotherapy (CRT) and radical surgery. It is unfortunate that we did not cover their experimental study, because they also revealed that Hh signalling activation mechanism is partly dependent on the ATP-binding cassette transporter family and that chemoresistance can be overcome by inhibitors of Hh pathways (Sims-Mourtada *et al*, 2007). Their papers have revealed the basic mechanism involved from experimental study seeking to overcome CRT resistance; whereas, we looked at the issue from a clinical perspective focusing on metastasis. In our paper, we have shown that persistent Gli-1 nuclear expression after CRT can predict very much earlier recurrence and poorer prognosis in ESCC patients; thus this a potential diagnostic biomarker and therapeutic target for ‘more aggressive’ cancer cells that can initiate relapse and maintain disease (Yoshikawa *et al*, 2008). This is of importance in clinical management, because high-risk patients can easily be screened by Gli-1 evaluation. Management of metastasis has been an important challenge to us, although since 1996 neoadjuvant CRT has shown improved resectability and a better prognosis in ESCC (Fujiwara *et al*, 2005). Hedgehog pathway antagonists have already been studied in phase I clinical trials in advanced or metastatic skin basal cell carcinomas. A combination modality of conventional anti-cancer agents plus Hh pathway antagonists could potentially abrogate both primary ‘bulk’ tumours and metastases in solid tumours (Feldmann *et al*, 2007).

It was pointed out that we did not carry out multivariate analysis. This was because the number of parameters was more than one-tenth the number of patients included in this study; therefore, we considered that a multivariate analysis might result in misleading conclusions. Thus, we performed a univariate analysis because of the small number of patients available for this study. Our findings will need to be confirmed in a larger more detailed study using multivariate analysis.

## References

[bib1] Feldmann G, Dhara S, Fendrich V, Bedja D, Beaty R, Mullendore M, Karikari C, Alvarez H, Iacobuzio-Donahue C, Jimeno A, Gabrielson KL, Matsui W, Maitra A (2007) Blockade of hedgehog signaling inhibits pancreatic cancer invasion and metastases: a new paradigm for combination therapy in solid cancers. Cancer Res 67: 2187–2196 17332349 10.1158/0008-5472.CAN-06-3281 PMC3073370

[bib2] Fujiwara Y, Kamikonya N, Inoue T, Koishi K, Yoshikawa R, Nakao K, Yagyu R, Nishiwaki M, Fujiwara M, Kojima S, Nakagawa K, Yamamura T (2005) Chemoradiotherapy for T3 and T4 squamous cell carcinoma of the esophagus using low-dose FP and radiation: a preliminary report. Oncol Rep 14: 1177–1182 16211282

[bib3] Sims-Mourtada J, Izzo JG, Ajani J, Chao KS (2007) Sonic Hedgehog promotes multiple drug resistance by regulation of drug transport. Oncogene 26: 5674–5679 17353904 10.1038/sj.onc.1210356

[bib4] Sims-Mourtada J, Izzo JG, Apisarnthanarax S, Wu TT, Malhotra U, Luthra R, Liao Z, Komaki R, van der Kogel A, Ajani J, Chao KS (2006) Hedgehog: an attribute to tumor regrowth after chemoradiotherapy and a target to improve radiation response. Clin Cancer Res 12: 6565–6572 17085672 10.1158/1078-0432.CCR-06-0176

[bib5] Yoshikawa R, Nakano Y, Tao L, Koishi K, Matsumoto T, Sasako M, Tsujimura T, Hashimoto-Tamaoki T, Fujiwara Y (2008) Hedgehog signal activation in oesophageal cancer patients undergoing neoadjuvant chemoradiotherapy. Br J Cancer 98: 1670–1674 18475300 10.1038/sj.bjc.6604361 PMC2391133

